# Subarachnoid hemorrhage secondary to cerebral venous sinus thrombosis

**DOI:** 10.1002/ccr3.1335

**Published:** 2018-03-03

**Authors:** Ashraf Abbas, Vijay Sawlani, Akram A. Hosseini

**Affiliations:** ^1^ Department of Medicine Nottingham University Hospitals NHS Trust Nottingham UK; ^2^ Department of Radiology University Hospitals Birmingham NHS Foundation Trust Birmingham UK; ^3^ Department of Neurology Queen's Medical Campus Nottingham University Hospitals NHS Trust Nottingham UK

**Keywords:** Cerebral venous sinus thrombosis, headache, subarachnoid hemorrhage

## Abstract

Subarachnoid hemorrhage as a presentation of cerebral venous sinus thrombosis (CVST) is a rare but recognized phenomenon. A high index of suspicion among clinicians and an awareness of subtle CT features can avoid delayed diagnosis of underlying CVST [Eur J Neurol., 17, 2010, 1249]. Prompt but careful anticoagulation can prevent significant associated morbidity and mortality.

A previously well 58‐year‐old man was admitted with deteriorating level of consciousness a day after profuse diarrhea and vomiting secondary to food poisoning. He scored 9/15 on the Glasgow Coma Scale, and in the right upper and lower limbs, there was no withdrawal from painful stimuli. He had bilateral papilledema but no neck stiffness. Three generalized tonic–clonic seizures were witnessed and terminated with intravenous phenytoin and levetiracetam.

Urgent noncontrast CT of his head reported subarachnoid hemorrhage in the left temporal and parietal lobes (Fig. 1A). The straight and superior sagittal venous sinuses appeared hyperdense prompting concerns of underlying venous thrombosis. A CT venogram demonstrated filling defects in the above‐mentioned sinuses confirming extensive cerebral venous sinus thrombosis (CVST) (Fig. 1B). Treatment was initiated with low molecular weight heparin, and he was later switched to rivaroxaban.

**Figure 1:**
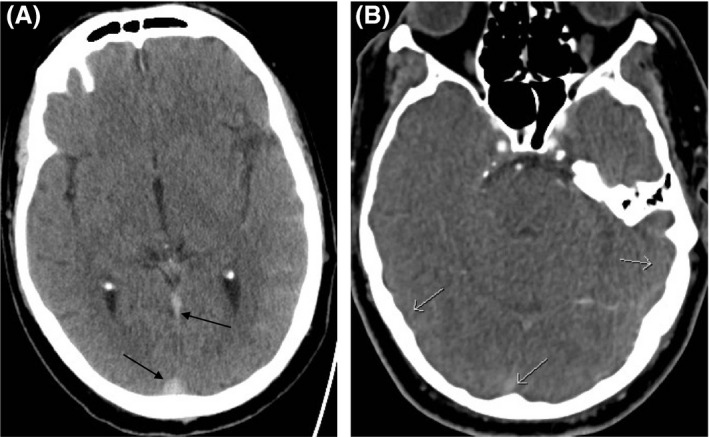
(A) Noncontrast‐enhanced CT shows subarachnoid hemorrhage in left temporal and parietal lobes and hyperdense thrombus in straight and superior sagittal venous sinuses (arrows). (B) CT venogram shows no opacification of superior sagittal sinus (arrow) or bilateral transverse sinuses (arrow), confirming venous sinus thrombosis.

Subarachnoid hemorrhage as a presentation of and secondary to CVST is a rare but recognized phenomenon 1. The distribution of localized subarachnoid hemorrhage sparing the basal cisterns and confined to the parasagittal or dorsolateral cerebral convexities has been described as another sign in noncontrast scans 2. CVST is associated with death rates between 5 and 30% 2. Prompt but careful anticoagulation is a life‐saving intervention.

## Authorship

AA: was a foundation year two doctor, wrote, and reviewed the literature. VS: was a consultant neuroradiologist and performed the image selection and analysis. AH: was a acting consultant neurologist and provided clinical details and manuscript editing.

## Conflict of Interest

None declared.
